# Response: “Cost‐effectiveness and budget impact analysis of the implementation of differentiated service delivery models for HIV treatment in Mozambique: a modelling study”: resource reductions are not equal to cost savings

**DOI:** 10.1002/jia2.26368

**Published:** 2024-10-15

**Authors:** Dorlim Moiana Uetela, Marita Zimmermann, Ruanne Barnabas, Kenneth Sherr

**Affiliations:** ^1^ Instituto Nacional de Saúde Marracuene Mozambique; ^2^ The Comparative Health Outcomes, Policy, and Economics Institute University of Washington Seattle Washington USA; ^3^ Division of Infectious Diseases Massachusetts General Hospital Harvard Medical School Boston Massachusetts USA; ^4^ Department of Global Health University of Washington Seattle Washington USA; ^5^ Department of Epidemiology University of Washington Seattle Washington USA; ^6^ Department of Industrial and Systems Engineering University of Washington Seattle Washington USA

1

Dear Editor,

We appreciate the opportunity to respond to the comments made in the letter “Cost‐Effectiveness and Budget Impact Analysis of the Implementation of Differentiated Service Delivery Models for HIV Treatment in Mozambique: a Modelling Study”: Resource reductions are not equal to cost savings [1].

First, we appreciate the authors’ recognition of the challenging work that we have done to generate evidence of the cost‐effectiveness and budget impact of differentiated service delivery (DSD) models for HIV treatment in Mozambique.

Second, we agree with the authors that the savings mentioned in our work are not monetary, but opportunity costs, mainly due to the reduction in the use of healthcare provider time. This reduction could theoretically allow providers to see more clients and/or provide higher‐quality care. The authors state that they have never encountered a healthcare system in sub‐Saharan Africa that is either able or willing to reduce its total complement of healthcare workers in response to the advent of DSD models, and no mechanism or pathway exists for DSD models to “save money.” We agree that these responses are unlikely. Rather, the reduction of provider time represents time that could be used to increase care for other clients or health areas, improving the health of the population overall without increasing costs. Our study focused on describing the opportunity costs saved through DSD model implementation. While investigating specifically how those savings could be used to advance health was beyond the scope of our work, we appreciate the discussion of implications and application of our work.

## COMPETING INTERESTS

The authors have no conflicts of interest to declare.

## AUTHORS’ CONTRIBUTIONS

DMU drafted the response. MZ, RB and KS reviewed the draft. All authors from the original article approved the final letter.

## Data Availability

Data underlying the results reported in this article will be made available at the request of investigators whose proposed use of the data has been approved by an independent review committee identified for this purpose. Request submissions should be sent online at https://ins.gov.mz/institucional/unidade-organicas/direccoes/directora-de-inqueritos-e-observacao-de-saude/solicitacao-de-dados/, and a data access agreement will need to be assigned per the procedures of Instituto Nacional de Saúde.
